# Glycogen Storage Disease: Expert Opinion on Clinical Diagnosis Revisited after Molecular Testing

**DOI:** 10.3390/genes14122219

**Published:** 2023-12-15

**Authors:** Rafael de Marchi, Tatiele Nalin, Fernanda Sperb-Ludwig, Franciele Cabral Pinheiro, Ida Vanessa Doederlein Schwartz, Carlos Eduardo Steiner

**Affiliations:** 1 Genética Médica e Medicina Genômica, Departamento de Medicina Translacional, Faculdade de Ciências Médicas, Universidade Estadual de Campinas (Unicamp), Campinas 13083-970, SP, Brazil; demarchi.rafael@gmail.com; 2 Departamento de Genética, Universidade Federal do Rio Grande do Sul (UFRGS), Porto Alegre 90010-150, RS, Brazil; tatinalin@gmail.com (T.N.); fcpbio@gmail.com (F.C.P.); idadschwartz@gmail.com (I.V.D.S.); 3 Laboratório BRAIN, Hospital de Clínicas de Porto Alegre, Porto Alegre 90035-903, RS, Brazil; 4 Programa de Pós-Graduação em Genética e Biologia Molecular, Universidade Federal do Rio Grande do Sul (UFRGS), Porto Alegre 90010-150, RS, Brazil

**Keywords:** inherited errors of metabolism, Glycogen Storage Disease, next-generation sequencing, clinical manifestations, clinical diagnosis, expert opinion, reverse phenotyping

## Abstract

This study sought to analyze whether an accurate diagnosis of the type and subtype of hepatic Glycogen Storage Diseases (GSDs) could be performed based on general clinical and biochemical aspects via comparing the proposed diagnostic hypotheses with the molecular results. Twelve physicians with experience in hepatic GSDs reviewed 45 real cases comprising a standardized summary of clinical and laboratory data. There was no relation between the hit rate and the time since graduation, the time of experience in GSD, and the number of patients treated during their careers. The average assertiveness was 47%, with GSD Ia and Ib being the best-identified types, while no expert correctly identified GSD IXc. Underage investigation for later manifestations, incomplete clinical description, and complementary analysis, the overvaluation of a specific clinical finding (“false positive”) or the discarding of the diagnosis in the absence of it (“false negative”), as well as the lack of knowledge of the rarest GSD types, may have impacted the accuracy of the assessment. This study emphasized that characteristics considered as determinants in identifying the specific types or subtypes of GSD are not exclusive, thus becoming factors that may have induced the evaluators to misdiagnose.

## 1. Introduction

Glycogen is the first energy source between meals and is especially abundant in the liver and muscles. In the liver, it serves as a glucose reserve for maintaining normoglycemia during fasting. Several enzymes act in the synthesis and degradation of glycogen, and a deficiency in any of these enzymes results in Glycogen Storage Diseases (GSDs), previously called glycogenosis, which causes abnormalities in the storage or use of glycogen [1,2].

GSDs comprise a group of inborn errors of metabolism (IEM) due to dysfunctions in glycogen metabolism causing multisystemic diseases that can present at any age from the neonatal period to adulthood [1,2,3,4,5,6]. The characteristics of GSDs depend on the abnormal site of glycogen metabolism. They are classified into numerical types that follow the historical order of their description, a system still widely used, at least until type VII [2]. Recent reviews suggest 20 types and subtypes of GSDs, divided into hepatic, muscular, mixed (hepatomuscular), and others limited to specific systems (two cardiac forms and one cerebral). The estimated GSD incidence is 1 case per 20,000–43,000 live births, and 80% of hepatic GSDs are caused by types I, III, and IX [4,7,8]. A summary of those with liver involvement is presented in Table 1.

The first step in the diagnosis of GSDs is a characterization of the patient’s phenotype and clinical features [5]. Although GSDs share similar clinical features to some extent, there is a wide spectrum of the phenotypic disease continuum [4,5]. The resulting phenotypic classes for each type of GSD can be used to aid diagnosis and treatment. These classes include hepatomegaly, hypoglycemia, intermittent myalgia, rhabdomyolysis, and hemolytic anemia [3]. Thus, the diagnosis requires carefully investigating the patient’s medical history [9]. Some metabolites inform the differential diagnosis of GSDs and can be useful for disease surveillance, including the measures of glucose, ketones, blood lactate, serum lipids and triglycerides, liver transaminases, uric acid, creatine kinase, and neutrophils count [5]. Hypoglycemia is the hallmark of hepatic GSDs, and hepatomegaly is a cardinal manifestation of GSDs with liver involvement, except for GSD 0 [4].

Historically, hepatic GSDs used to be confirmed with histological analysis via liver biopsy [2], which should lead to a definitive diagnosis in most cases but are critically dependent on correct tissue processing [10]. In recent years, invasive and technique-dependent exams, not always available in all services, have been progressively replaced by molecular studies in DNA extracted from peripheral blood lymphocytes or oral mucosa swabs, given the availability of target gene sequencing or multigenic panels, with the advantages of being a less invasive option for confirmatory diagnosis and often shortening the diagnostic odyssey [5,6,10]. Therefore, the definitive diagnosis is a combination of clinical presentation, biochemical abnormalities, imaging, histology, the determination of enzyme activity in liver tissue, and molecular genetic analysis [5,9]. The limitations of genetic testing include the possibility of unidentified variants in tested genes and the individual’s symptoms having an etiology unrelated to variants in the genes tested [5,6].

## 2. Materials and Methods

The current project sought to verify, analyze, and discuss whether an accurate diagnosis of the type and subtype of hepatic GSD could be performed on general clinical and biochemical aspects, proposing the analysis of real cases with different GSDs confirmed with molecular tests (NGS gene panel) previously studied by our group (Supplementary Table S1) [8]. The research design included (1) a selection of physicians with clinical experience in GSDs, (2) a selection and summary of 45 real cases transcribed in forms containing the clinical and laboratory data available at the time samples were sent to molecular analysis, and (3) an evaluation of the cases by the experts asking for diagnostic hypotheses, followed by feedback on the type and subtype after the molecular confirmation and free comments on their correct or incorrect answers.

### 2.1. Selection and Consent of Research Subjects

A non-probabilistic sample was established to include 15 subjects (also called “experts”) to obtain relevant information and generate the necessary analyses to complete this research. Inclusion criteria were medical degree and previous experience in GSDs. Once a multidisciplinary team managed GSD, recruiting subjects from different specialties to compose the group of experts was considered relevant. Invitation letters and written informed consent were sent to the participants after institutional ethical approval (protocol CAAE 23072119.3.0000.5404, CEP/FCM/Unicamp).

Seventeen physicians with previous experience in hepatic GSDs were invited to participate in this project; two declined the invitation, one confirmed participation via email but did not sign the consent, and 14 formally consented and were included in this study. Two of the 14 consenting participants did not fill out the forms, resulting in 12 experts who evaluated the proposed cases.

Recruitment was terminated once it was observed that GSD is a rare disease, the number of experts in Brazil is quite limited, and the recruited group included different specialties, distinct lengths of experience, and representatives from different states and prominent institutions in the country.

### 2.2. Definition of Clinical Cases and Randomization

In an anonymized manner, clinical, biochemical, and genetic data were extracted from 45 cases of a previous study [8]. Gender, age, current symptoms, and the results of biochemical tests, among others, were used, as reported voluntarily by the attending physicians, to prepare the clinical cases evaluated by the experts (Supplementary Table S2). Data reproduced the physicians’ actual clinical and laboratory evaluations until samples were sent for molecular testing.

The 45 cases were divided into three groups, balanced based on (1) the percentage of completion of clinical forms, (2) the presence of comments about the case, and (3) card numbering order, establishing the number of 15 patients per group. The case group allocation for each expert was performed manually, sequentially, and followed the chronological order of signature of the consent form, resulting in the distribution of interspersed groups for each expert in ascending order (groups 1, 2, and 3, respectively).

### 2.3. Evaluation of Clinical Cases

All cases were analyzed separately by at least three different experts. The responses of each expert were compared with the genetic result to establish the accuracy and the percentage of correspondence between phenotype and genetic basis. In the second step, each expert was given feedback on their correct and incorrect answers and was asked to comment on their assertiveness (free text question).

## 3. Results

### 3.1. General Analysis of the Group of Experts

Most participants (8/12, 67%) reported 20 or more years of medical degree. The distribution among specialties was six (50%) geneticists, four (33%) gastroenterologists/hepatologists, one (8.5%) neurologist, and one (8.5%) nephrologist. Of the 12 participants, seven (58%) reported being specialists in pediatrics as well.

GSD experience of “0–5 years” and “16–20 years” was reported by three (25%) experts each; experience of “6–10 years”, “11–15 years”, and “20+ years” was reported by two (17%) experts each. Most participants, 11/12 (92%), reported having an “intermediate” level of knowledge in GSD, and one (8%) self-declared “advanced” knowledge in GSD.

Six (50%) experts reported having followed “11 to 20 patients” with GSDs during their career, followed by three (25%) experts who experienced “0 to 10 patients”, two (17%) experts who experienced “50+ patients”, and one (8%) that experienced “0 to 10 patients” in their careers. GSD Ia was reported as the most common type seen in clinical practice (100%). “Public hospitals” represented the principal place of professional practice for eight (67%) of the experts, and seven (58%) experts reported working in Reference Services for Rare Diseases. All 12 experts (100%) reported “university institutions” as the main place of professional practice, where most patients are known to be concentrated.

### 3.2. General Characteristics of the Clinical Cases

When conducting the general analysis of the clinical cases, the male-to-female sex ratio was 2:1, and parental consanguinity was reported in eight (18%) cases. The mean age of patients at diagnosis was two years, with a median of eight months, and the mean age of onset of symptoms was seven months, with a median of two months.

Seven of the 10 types of hepatic or hepatomuscular GSD were present in the sample, the most common being GSD Ia with 19 patients (42%), followed by GSD Ib with seven patients (16%), and GSD IXa with six patients (13%); the less frequent types in this sample were GSD III (*n* = 5; 11%), GSD IXb (*n* = 4; 9%), GSD IXc (*n* = 3; 7%), and GSD VI (*n* = 1; 2%) (Supplementary Table S1). There were no cases of GSD 0a, IV, or FBS.

**Figure 1:**
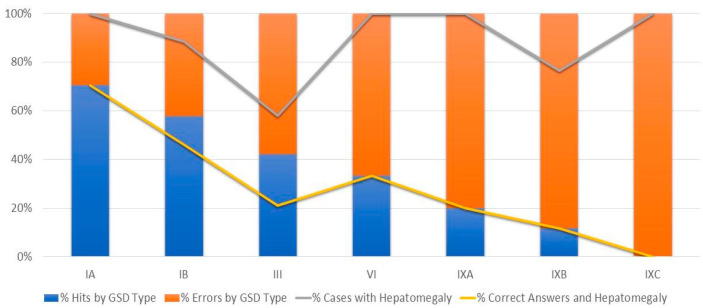
Percentage of correct answers in the presence of hepatomegaly.

**Figure 2:**
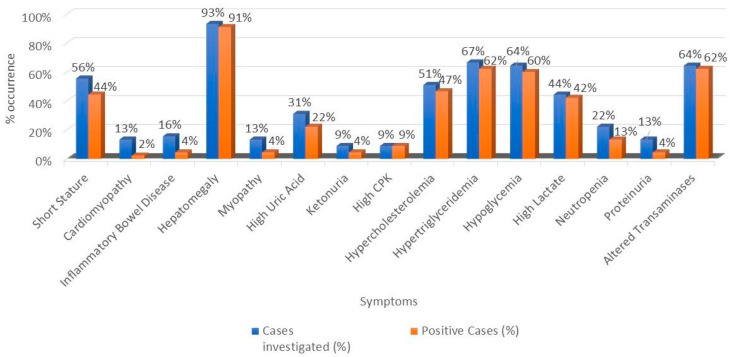
Frequency of clinical and laboratory findings and their positivity rate.

The most frequent clinical symptom was hepatomegaly (93%) (Figure 1), followed by short stature (56%); inflammatory bowel disease, cardiomyopathy, and myopathy were investigated in approximately 15% of patients and were negative in most cases (Figure 2).

Considering the data contained in the clinical files, the most investigated biochemical features were hypertriglyceridemia (67%), transaminase alteration (64%), and hypoglycemia (64%) (Figure 2), all positive in more than 90% of the investigated cases. Ketonuria and CPK levels were the least investigated features (only in four patients); however, CPK elevation was confirmed in 100% of the circumstances in which it was investigated.

### 3.3. Assertiveness of Clinical Cases

Each of the 12 experts analyzed 15 cases, totaling 180 evaluations. The average of correct answers by the experts was 47%, with a median of 40%, with the highest individual hit rate of 73% and the lowest of 33%.

### 3.4. Assertiveness by Type of GSD

Six of the 180 evaluations were disregarded for this analysis because they were blank, or more than one alternative was selected; the final analysis was based on the total number of 174 forms.

The most recognized GSD type by the experts was GSD Ia, with an average hit rate of 71%, followed by Ib with 58% of hits, while the least recognized was the GSD IXc, which was not correctly identified by any of the experts (Table 2).

### 3.5. Feedback from the Experts

Of the 36 feedbacks from the experts, 22 (61%) dealt with cases that did not have the correct hypothesis indicated, half of them (11 cases, 50%) were attributed to the lack of clinical and biochemical data provided in the forms, five (23%) to the presence of confounding factors, four (18%) reported being compatible differential diagnoses that make difficult to differentiate based only on clinical and biochemical symptoms, one case justified by rarity (GSD VI), and another case by the lack of experience with the specific subtype (GSD IXa).

**Figure 3:**
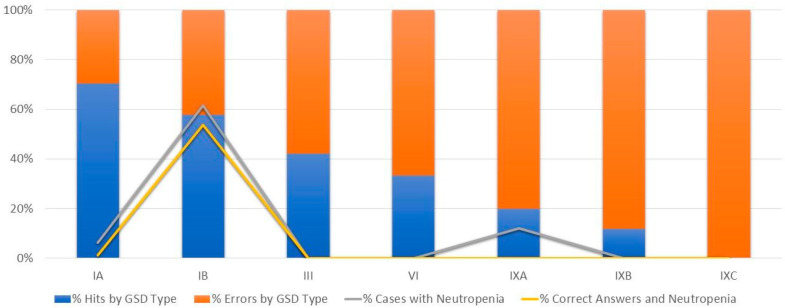
Percentage of correct answers in the presence of neutropenia.

Of the 14 responses received for the cases of correct diagnosis, 10 (71%) of them attribute the correct answer to findings that determine or suggest the type and subtype of GSD, for example, neutropenia (Figure 3). Despite having proposed the correct diagnostic hypothesis, the other four (29%) commented cases mentioned the difficulty of analysis due to lack of data and the presence of confounding factors.

## 4. Discussion

This study aimed to analyze whether the diagnosis of a specific GSD type could be made by the referring physicians on their clinical praxis. Therefore, cases were based on actual investigations instead of the construction of didactic examples. Thus, although theoretically some of the types and subtypes of GSD could be distinguished with a complete clinical and biochemical investigation, actual praxis showed that data collection tends to be incomplete, including missing biochemical data in some cases, besides the absence of other valuable tests for the diagnosis of hepatic GSDs such as glucose tolerance test or fasting blood glucose-lactate relationship and CT liver imaging. This shows the heterogeneity of investigation routines among physicians or medical specialties and the lack of laboratory support, even in centers for rare diseases. Moreover, a long and invasive investigation, including full biochemical analysis and hepatic biopsy for histological and/or enzymatic studies, seems to be replaced when a single less invasive testing approach (for example, oral swabs) based on molecular analysis is available as a first option test.

Regarding the group of experts, the relation between the hit rate and the time since graduation in medicine, the time of experience in GSD, and expertise in the number of patients with GSD treated during their careers did not present a linear result, making it impossible to assess the impact of these characteristics on diagnostic accuracy.

Given that the average assertiveness of the clinical forms was 47%, when analyzing the relation between the fill percentage and the average hit rate, it was observed that cases with less than 50% of data filling (135 forms) had an average accuracy of 43%, while patients with 50% of data filling or more (45 forms) had an average accuracy of 58%, representing a difference of 15 percentage points, favoring the identification of cases with data completion more than 50%.

Of the 45 cases, 21 (47%) had a percentage of correct answers greater than or equal to 50%, and 12 (27%) were correctly identified by all experts. Of the 24 (53%) cases with less than 50% accuracy, 14 (31%) were incorrectly identified by experts.

An average data filling of 29% was observed in cases not correctly identified by any experts, compared to an intermediate data filling of 38% in cases correctly identified by all experts. However, it is noted that the data filling was higher in the group that had between 1% and 49% accuracy. In this analysis, it is argued that despite the nine-percentage points difference in data filling between the cases that obtained 100% correct answers and 100% errors, it is not possible to create a direct relation between the data completion and the accurate diagnosis.

Despite the assertiveness analysis of the clinical cases not indicating a consistent relation between the data filling and accuracy, the availability of clinical and biochemical data reported in the patients were the main points of attention raised by the experts during the feedback. The experts’ feedback reinforces the possibility that determining factors that were inadequately investigated may have impacted the accuracy of the assessment.

When evaluating the cases with 100% correct and 100% incorrect evaluation and the comparison with the types of GSDs, it was noted that the GSD Ia stood out as the type best identified by the experts, followed by the GSD Ib. At the same time, GSD IX has the most significant number of unidentified cases, either because of greater knowledge reported by experts about GSD Ia or because of the lack of an investigation capable of differentiating this from other types. Theoretically, since an underlying metabolic pathway characterizes GSD I, it has the highest diagnostic probability based only on general clinical and biochemical aspects. In addition, because general clinical and biochemical aspects are shared by types IX and VI, which share an abnormality in the phosphorylase pathway, it is impossible to distinguish between them based on this information alone.

According to the assertiveness analysis by type and subtype, GSD Ia had the highest overall success rate per case and in all groups. This was also the type of GSD most present in the hypotheses and had the best accuracy when comparing the number of diagnostic hypotheses proposed with the hit rate.

Among the factors that may have influenced the clinical diagnosis by the specialist are the underage investigation for later manifestations of the disease, the clinical description, and incomplete complementary analysis, in addition to the overvaluation of a particular clinical finding (“false positive”) or the discarding the diagnosis in the absence of it (“false negative”), as well as the lack of knowledge of the clinical type by the specialist.

In the present study, the mean age at diagnosis was nine months for types Ia and Ib, 24 months for type VI, 27 months for type IXa, and 108 months for type III. Investigation was below the age of onset of later symptoms, such as hyperuricemia (typically starting after puberty) or adenomas (after the 2nd or 3rd decade) in type Ia, neutropenia (up to 9 years of age), and chronic inflammatory disease (diagnosed at an average age of 8.7 years) in type Ib, or progression to cirrhosis in types III and IXa (also after puberty) [2,7,11]. Therefore, the expert interpretation of clinical data may be compromised by a lack of information regarding the natural history of each of these conditions.

Symptoms and signs such as hepatomegaly, hypertriglyceridemia, elevated liver transaminases levels, and hypoglycemia were widely investigated and were altered in more than 90% of the studied cases. These factors are relevant for diagnosing GSD; however, they are not determining factors for defining the subtype, as exemplified in the graph in Figure 1.

On the other hand, signs such as neutropenia and CPK elevation have been poorly investigated in the present sample. They could have been better used to identify the GSD subtype without specific genetic testing. Such information suggests that the biochemical investigation of these cases was incomplete, being unhelpful for the correct clinical diagnosis of two of the three most frequent forms of hepatic and hepatomuscular GSD, as demonstrated in the graph of Figure 3.

The overvaluation of a single clinical finding as strongly indicative of the disease when present, or discarding such a diagnosis when absent, may explain part of the low scores given by experts, such as neutropenia and chronic inflammatory bowel disease in type Ib. Similarly, the presence of progressive muscle symptoms (positive) or the absence of CPK elevation (false negative) may have made it difficult to identify cases with type III correctly.

The analysis of the experts’ proposed diagnostic hypotheses corroborates the statement, since of the seven cases diagnosed as GSD Ib by NGS, six were investigated for neutropenia, four positive and two negatives. Positive patients for neutropenia had an average accuracy of 90%, while negative cases for neutropenia had an average accuracy of only 15%. Some experts did not correctly identify the GSD Ib case without information on neutropenia. Likewise, two reports of neutropenia occurred in patients with subtypes Ia and IXa, with a mean accuracy of 10% (20% and 0%, respectively). These two cases add up to eight evaluations by the expert group, with seven mistakenly classified as GSD Ib.

CPK elevation was investigated and confirmed in four patients, but only two confirmed a final diagnosis of GSD III. The proportion of correct answers for the two cases of GSD III with CPK elevation was 90%, while the other two cases (Ia and IXa) were not correctly identified by any of the experts, with GSD III being the most common hypothesis in three of the seven evaluations. Another three GSD III cases confirmed via NGS were present in the project; however, without CPK investigation, the experts correctly identified none.

These same parameters were reaffirmed during the feedback analysis. Of the five cases in which the reason for an incorrect diagnostic hypothesis was attributed to confounding factors, the explanations refer to one case of elevated CPK, a characteristic suggestive of GSD III, but with a diagnosis confirmed via NGS of GSD Ia; two cases of neutropenia, a finding most suggestive of GSD Ib, however with a confirmed diagnosis of GSD Ia via NGS; and two patients with a confirmed diagnosis of GSD Ib, but without presenting neutropenia.

Indeed, the lack of a determining factor and the atypical presentations made it difficult for specialists in GSD to make an accurate diagnosis; however, just as relevant was the lack of knowledge of less frequent forms, such as GSD VI and GSD IX.

The only case of GSD VI present in this study was evaluated by three experts, being correctly identified by only one of them. Among the experts’ feedback, the only argument about this case justified the incorrect identification due to the rarity of this type of GSD and the absence of a specific clinical marker.

GSD IX is a less common type and presents subtypes following autosomal recessive or X-linked recessive inheritance, making its recognition more difficult without a clear family history. In the present study, patients with type IX had an accuracy rate of only 13%, compared to the overall average accuracy of 48%. It was also observed that, based on the nine cases of GSD IX that received feedback from the experts, in addition to the lack of data and professional experience with this specific type of GSD, the low prevalence and the similarity with other types of GSD were highlighted.

IEM are usually severe conditions, and the precise identification of the molecular basis of these diseases is essential for adequate patient treatment and genetic counseling [12]. In addition to reducing costs and optimizing time, in these situations, molecular confirmation is critical in clinical practice to (a) differentiate distinct subtypes in a group that encompasses several clinically overlapping conditions, (b) establish a correct diagnosis aiming at clinical follow-up and management measures [13], and (c) help in family planning and the genetic counseling of other family members [14,15]. Still, from the point of view of clinical research, it can serve to (d) describe new variants that cause or modulate the disease [8,16], (e) add helpful information in the discussion of the genotype–phenotype relationship and the reclassification of variants of uncertain significance [17], or even (f) identify new genes that cause similar clinical conditions [18].

## 5. Conclusions

It was observed in the present study that known characteristics such as hepatomegaly and hypoglycemia, typical symptoms of GSDs, are insufficient to determine the type and subtype, which makes a detailed investigation necessary when there is diagnostic suspicion. Without a suggestive factor characteristic of a specific type of GSD, such as neutropenia, neuromuscular symptoms, or a family history suggestive of X-linked inheritance, the most proposed diagnostic hypothesis was GSD Ia. This finding possibly occurs because it is the most prevalent and recognized type of GSD in clinical practice, which could cause GSD Ia to be the most frequent alternative with the highest success rate. However, characteristics considered determinants in identifying the specific types or subtypes of GSD, as cited above, are not exclusive, thus becoming factors that may have induced the experts to make a diagnostic error.

The experts also reinforced the importance of completing a full analysis during the feedback. However, only a modest increase in the correct identification of the type and subtype of GSD was observed when more clinical and biochemical data were available.

Another relevant factor is the moment of the clinical and biochemical evaluation of the natural history of the disease. The natural history of some types of GSD can lead to late symptoms that were not present at the time of diagnosis, which could have helped in a more correct diagnosis.

In addition to the conclusions presented, the study team aims, based on their most recent professional experience, to observe that not only in the specialized literature publications but also in clinical practice, NGS techniques are becoming increasingly cheaper and more accessible for experts and generalists, which has made its use increasingly frequent to the detriment of traditional single-target gene sequencing techniques or even biochemical tests as a first-line technique for diagnostic investigation. This reinforces a tendency for the diagnosis of these genetic conditions to migrate from the traditional sequence of “clinical evaluation followed by biochemical investigation followed by molecular confirmation” to the sequence “clinical evaluation followed by molecular investigation followed by biochemical confirmation (eventually ending in the second stage)” or, perhaps, even more paradoxical, to the sequence “molecular investigation then biochemical confirmation and finally clinical correlation”.

Given the challenges observed when seeking an accurate diagnosis based only on clinical symptoms and general biochemistry, this research proposes a paradigm shift via reinforcing the tendency to migrate from biochemical and enzymatic to molecular diagnosis due to its practicality, time, cost, and specificity. However, the importance of basic clinical assessment to improve clinical and laboratory data is critical for situations where molecular testing is inconclusive, such as interpreting variants of uncertain significance or the presence of a single variant in heterozygosity for a recessive condition; for these situations, functional tests might still be necessary to establish genotype–phenotype correlations. When laboratory data are previously collected in routine care, it reduces time and energy instead of patient recruitment for prospective data collection, such as in reverse phenotyping studies.

## Figures and Tables

**Table 1:** Genetic and epidemiologic characteristics of the hepatic GSDs [4,5,8].

Type	Gene	OMIM	Location	Enzyme Deficiency	Inheritance	Incidence
0a	*GYS2*	240600	12p12.1	Liver glycogen synthase	AR	-
Ia	*G6PC*	232200	17q21	Glucose-6-phosphatase	AR	1 in 50,000–100,000
Ib	*SLC37A4*	232220	11q23.3	Glucose-6-phosphate transporter	AR	-
III	*AGL*	232400	1p21.2	Glycogen debranching enzyme	AR	1 in 83,000–100,000
IV	*GBE1*	232500	3p12.3	Glycogen branching enzyme	AR	1 to 600,000–800,000
VI	*PYGL*	232700	14q22.1	Liver glycogen phosphorylase	AR	1 in 65,000–85,000
IXa	*PHKA2*	306000	Xp22.13	Phosphorylase kinase, α subunit	XL	1 in 100,000
IXb	*PHKB*	261750	16q12.1	Phosphorylase kinase, β subunit	AR	-
IXc	*PHKG2*	613027	16p11.2	Phosphorylase kinase, γ subunit	AR	-
FBS ^1^	*SLC2A2*	227810	3q26.2	Facilitated glucose transporter 2	AR	-
XII	*ALDOA*	611881	16q22-q24	Aldolase A ^2^	AR	-

Key: - = unavailable data; AR = autosomal recessive; XL = X-linked; ^1^ Fanconi–Bickel syndrome; ^2^ or Fructose 1,6-biphosphate Aldolase A.

**Table 2:** Hit rate by GSD type.

GSD Type	Forms (*n* = 180) % of Clinical Records	Diagnostic Suspicions (*n* = 174) % of Suspicions	Hit Rate
Ia	78 (54%)	81 (47%)	71%
Ib	26 (14%)	27 (16%)	58%
III	19 (11%)	26 (15%)	42%
VI	3 (2%)	17 (10%)	33%
IXa	25 (14%)	17 (10%)	20%
IXb	17 (9%)	5 (3%)	12%
IXc	12 (7%)	1 (1%)	0%

## Data Availability

The data supporting this study’s findings are available from the corresponding author upon reasonable request.

## Supplementary Materials

The following supporting information can be downloaded at: https://www.mdpi.com/article/10.3390/genes14122219/s1, Table S1: Genotype data of the patients/cases that based the clinical discussion; Table S2: Clinical Form (Template).
