# Supplementary material for: Glycogen Storage Disease: Expert Opinion on Clinical Diagnosis Revisited after Molecular Testing
Source: Genes (Basel). 2023 Dec 15;14(12):2219. doi: 10.3390/genes14122219 (PMC10743078; doi:10.3390/genes14122219)
Supplement: Supplementary file 1 [file genes-14-02219-s001.zip › genes-2705758-supplementary.pdf]

**Supplementary Table S1.** Genotype data of the patients/cases that based the clinical discussion [8].

| Case | | Gene | Codon | Protein | Zygosity | Classif. [1] | GSD Type |
|---|---|---|---|---|---|---|---|
| # | Sex | | | | | | |
| 1 | F | *PHKG2* | c.927+1G>A | p.? | hom | LP | IXc |
| 2 | M | *PHKA2* | c.3629G>A | p.Gly1210Glu | hem | LP | IXa |
| 3 | M | *G6PC* | c.247C>T | p.Arg83Cys | het | P | Ia |
| | | | c.231-1G>A | p.? | het | P | |
| 4 | M | *G6PC* | c.77delC | p.Ser26=fs | het | P | Ia |
| | | | c.563-3C>G | p.? | het | VUS | |
| 5 | M | *G6PC* | c.247C>T | p.Arg83Cys | hom | P | Ia |
| 6 | F | *PYGL* | c.697G>A | p.Gly233Ser | hom | P | VI |
| 7 | F | *G6PC* | c.563-3C>G | p.? | hom | VUS | Ia |
| 8 | F | *SLC37A4* | c.899G>A | p.Arg300His | het | P | Ib |
| | | | c.1042_1043delCT | p.Leu348Valfs*53 | het | P | |
| 9 | F | *PHKB* | c.1972-2A>G | p.? | het | P | IXb |
| | | | c.2060C>G | p.Ser687* | het | LP | |
| 10 | F | *AGL* | c.1383G>A | p.Trp461* | het | P | III |
| | | | c.3475_3476insA | p.Gln1159fs | het | LP | |
| 11 | M | *SLC37A4* | c.703_705delGTG | p.Val236del | het | P | Ib |
| | | | c.1042_1043delCT | p.Leu348Valfs*53 | het | P | |
| 12 | M | *G6PC* | c.247C>T | p.Arg83Cys | hom | P | Ia |
| 13 | M | *SLC37A4* | c.899G>A | p.Arg300His | hom | P | Ib |
| 14 | F | *AGL* | c.3214_3215delGA | p.Glu1072Aspfs | het | P | III |
| | | | c.4528_4529insA | p.Tyr1510Terfs | het | P | |
| 15 | M | *G6PC* | c.247C>T | p.Arg83Cys | het | P | Ia |
| | | | c.1039C>T | p.Gln347* | het | P | |
| 16 | F | *PHKB* | c.572_576delAGATT | p.Gln191Hisfs | het | LP | IXb |
| | | | c.1972-2A>G | p.? | het | P | |
| 17 | M | *G6PC* | c.247C>T | p.Arg83Cys | hom | P | Ia |
| 18 | M | *PHKB* | c.1972-2A>G | p.? | het | P | IXb |
| | | | c.2060C>G | p.Ser687* | het | LP | |
| 19 | M | *G6PC* | c.563-3C>G | p.? | het | VUS | Ia |
| | | | c.1039C>T | p.Gln347* | het | P | |
| 20 | M | *SLC37A4* | c.1042_1043delCT | p.Leu348Valfs*53 | hom | P | Ib |
| 21 | M | *G6PC* | c.247C>T | p.Arg83Cys | hom | P | Ia |
| 22 | F | *G6PC* | c.70C>T | p.Gln24* | het | P | Ia |
| | | | c.161A>C | p.Gln54Pro | het | P | |
| 23 | M | *G6PC* | c.70C>T | p.Gln24* | het | P | Ia |
| | | | c.161A>C | p.Gln54Pro | het | P | |
| 24 | F | *G6PC* | c.247C>T | p.Arg83Cys | het | P | Ia |
| | | | c.401_402delCT | p.Thr134=fs | het | P | |
| 25 | F | *G6PC* | c.247C>T | p.Arg83Cys | het | P | Ia |
| | | | c.401_402delCT | p.Thr134=fs | het | P | |
| 26 | F | *SLC37A4* | c.1042_1043delCT | p.Leu348Valfs*53 | hom | P | Ib |
| 27 | M | *SLC37A4* | c.1042_1043delCT | p.Leu348Valfs*53 | hom | P | Ib |
| 28 | M | *G6PC* | c.247C>T | p.Arg83Cys | het | P | Ia |
| | | | c.161A>C | p.Gln54Pro | het | P | |

**Supplementary Table S1.** continuation.

| Case | | Gene | Codon | Protein | Zygosity | Classif. [1] | GSD type |
|---|---|---|---|---|---|---|---|
| # | Sex | | | | | | |
| 29 | M | *PHKB* | c.1972-2A>G | p.? | het | P | IXb |
| | | | c.2181delT | p.Leu728Phefs*4 | het | LP | |
| 30 | M | *PHKA2* | c.883C>T | p.Arg295Cys | hem | P | IXa |
| 31 | M | *PHKA2* | c.883C>T | p.Arg295Cys | hem | P | IXa |
| 32 | M | *PHKA2* | c.2452C>T | p.Gln818* | hem | LP | IXa |
| 33 | M | *G6PC* | c.247C>T | p.Arg83Cys | hom | P | Ia |
| 34 | M | *G6PC* | c.247C>T | p.Arg83Cys | het | P | Ia |
| | | | c.1039C>T | p.Gln347* | het | P | |
| 35 | M | *AGL* | c.1571G>A | p.Arg524His | het | P | III |
| | | | c.3625C>T | p.Gln1209* | het | LP | |
| 36 | M | *AGL* | c.744G>A | p.Trp248* | het | LP | III |
| | | | c.3484C>T | p.Gln1162* | het | P | |
| 37 | M | *SLC37A4* | c.706_708delGTG | p.Val236del | het | P | Ib |
| | | | c.1042_1043delCT | p.Leu348Valfs*53 | het | P | |
| 38 | F | *G6PC* | c.202G>A | p.Gly68Arg | het | P | Ia |
| | | | c.439A>T | p.Arg147* | het | LP | |
| 39 | M | *PHKG2* | c.502C>T | p.Arg168* | hom | P | IXc |
| 40 | M | *AGL* | c.293+2T>A | p.? | het | LP | III |
| | | | c.1858_1859delCT | p.Leu620Valfs*7 | het | P | |
| 41 | F | *PHKG2* | c.835C>T | p.Arg279Cys | het | LP | IXc |
| | | | c.927+1G>A | p.? | het | LP | |
| 42 | M | *PHKA2* | c.3614C>T | p.Pro1205Leu | hem | P | IXa |
| 43 | F | *G6PC* | c.809G>T | p.Gly270Val | hom | P | Ia |
| 44 | M | *PHKA2* | c.883C>T | p.Arg295Cys | hem | P | IXa |
| 45 | M | *G6PC* | c.161A>C | p.Gln54Pro | hom | P | Ia |

Obs.: Patients 9 and 18, 22 and 23, and 24 and 25 are pairs of siblings.

Key: F = female; hem = hemizygous; het = heterozygous; hom = homozygous; LP = likely pathogenic; M = male; P = pathogenic; VUS = variant of unknow significance.

[1] The variant classification following ACMG criteria was updated on Dec. 12th, 2023; asserted at the Franklin by Genoox Database (https://franklin.genoox.com/clinical-db/home).

**Supplementary Table S2. Clinical Form (Template).**

| | | |
|---|---|---|
| **Case #: XX** | | Filling Date:___/___/_____ |
| Age: | Sex: (   ) F   /  (   ) M | Parental consanguinity: (   ) Y  /  (   ) N |
| Age of onset of symptoms: | | |
| Uric acid: | | |
| Liver biopsy: | | |
| Ketonuria: | | |
| Cholesterol level: | | |
| CPK: | | |
| Inflammatory bowel disease: | | |
| Hepatomegaly: | | |
| Hypoglycemia: | | |
| Lactate: | | |
| Neutropenia: | | |
| Proteinuria: | | |
| Transaminases: | | |
| Triglycerides: | | |

| |
|---|
| In your opinion, the clinical case described is compatible with which type of glycogenosis?<br>(   )Ia   (   )Ib   (   )III   (   )VI   (   )IXa   (   )IXb   (   )IXc |
| Comments: |
